# Experimental Investigations Concerning Suppuration

**Published:** 1890-10

**Authors:** 


### Experimental Investigations Concerning Suppuration.

In a recent thesis Dr. J. de C. Holmfeld discusses the causation of suppuration and reaches the following conclusions: In all cases of warm abscesses in men submitted to bacteriological examination the existence of micro-organisms in the pus has been demonstrated. These micro-organisms are few in number and well marked. Rosenbach has described them under the following names: Staphylococcus pyogenes aureus, staphylococcus pyogenes albus, micrococcus pyogenes tennis, streptococcus pyogenes. But besides the suppuration due to the presence of microbes, we are able to produce in animals suppuration that is purely chemical and entirely independent of microbes. The author has inoculated, with the greatest antiseptic precautions, the following substances: Essence of turpentine, petroleum, chloride of zinc in 10 per cent. solution, glycerine, and nitrate of silver in 5 per cent solution. These substances, which produce no appreciable effect upon the rabbit, provoke considerable suppuration in dogs. The microscope discovers not the slightest trace of micro-organisms in this pus; bouillon and gelatine remain absolutely sterile. These experiments prove that the suppuration is by no means dependent upon the presence of micro-organisms it the tissues. The author therefore raises the question whether the suppurative action of the pyogenic micro-organisms in the tissues is not dependent upon the presence of irritating substances in the products of secretion or in the bodies of the microbes themselves, for we are able to extract from the cultures and from the body of the staphylococcus aureus several chemical substances which are capable of producing a very pronounced pyogenic effect. The author concludes that acute suppuration should be considered as the result of chemical influences upon the organism.—*Gaz. Med. de Paris.*

